# A task-aligned multimodal machine learning framework for studying working memory dysfunction in Parkinson’s disease

**DOI:** 10.1007/s11571-026-10526-z

**Published:** 2026-08-13

**Authors:** Mercedes Terry, Samuel A. Birkholz, Jeffery S. Johnson, Jau-Shin Lou, Asenath X. Heuther, Jessica Keller, Enrique Alvarez Vazquez

**Affiliations:** 1 Biomedical Engineering Department, University of North Dakota, Grand Forks, ND 58202 USA; 2 Department of Psychology, North Dakota State University, Fargo, ND 58102 USA; 3 Department of Neurology, Sanford Health, Fargo, ND 58103 USA; 4 Department of Neurology, University of North Dakota School of Medicine and Health Science, Grand Forks, ND 58202 USA; 5 Department of Psychology, Minnesota State Community and Technical College, Moorhead, MN 56560 USA

**Keywords:** Parkinson’s disease, Working memory, Electroencephalography, Feature engineering, Principal component analysis, Machine learning

## Abstract

Cognitive impairment is one of the most functionally debilitating non-motor symptoms in Parkinson's disease (PD). Yet, current diagnostic and clinical practices rely heavily on subjective assessments and burdensome behavioral testing, which lack the temporal resolution to detect subtle deficits. While EEG and pupillometry offer promising non-invasive insights into cognitive processing, more scalable and objective methods are needed to detect subtle and often elusive dysfunction in early PD. Machine learning (ML) leverages patterns in neurophysiological signals recorded during cognitive tasks to identify subtle impairments that may not be evident in standard evaluations. We recorded EEG and pupillometry data from 68 participants (35 PD, 33 healthy controls (HC)) during a visual Change Detection working memory task. Using our custom, standardized feature-extraction toolbox, we extracted 108 features and incorporated the resulting feature matrices into an ML pipeline. We applied SMOTE to balance the classes, then used principal component analysis (PCA) to reduce dimensionality. An automated elbow method identified optimal cutoffs for principal components (PCs) and original features, which guided subsequent recursive elimination (RE) for computational efficiency. We trained a support vector machine with a radial basis function kernel (SVM-RBF) to classify PD and evaluated the final model's performance on a hold-out dataset. The automated elbow method identified optimal cutoffs at 20 PCs and 22 original features. The RE revealed that a model using 14 PCs and the top 7 PCA-weighted features achieved the highest performance, with 71% accuracy and an F1 score of 0.701. The classifier's performance declined slightly when tested on held-out data, with an accuracy of 63% and an F1 score of 0.626. This study demonstrates the potential of a task-aligned ML pipeline for uncovering early cognitive markers of PD. The framework supports post-hoc feature contribution mapping, linking retained physiological features and task stages back to classification-relevant patterns, supporting mechanistic investigation of cognitive dysfunction in PD.

**Supplementary Information:**

The online version contains supplementary material available at 10.1007/s11571-026-10526-z.

## Introduction

Parkinson’s disease (PD) is a progressive neurodegenerative disorder traditionally defined by motor symptoms such as tremor, rigidity, and bradykinesia (Armstrong and Okun 2020). Yet cognitive impairments also emerge early and substantially affect daily functioning and quality of life. Despite their predictive value for decline and progression, these non-motor symptoms are often overlooked and undertreated (Armstrong and Okun 2020; Gerner et al. 2025). Working memory (WM) is one of the most consistently affected domains, supporting reasoning, decision-making, and goal-directed behavior as well as attention and executive control (Baddeley 1992; Fang et al. 2024). In PD, WM deficits in updating, attentional filtering, and storage capacity are linked to frontostriatal dysfunction. They are especially evident in visual WM tasks that manipulate cognitive load, where patients perform worse than healthy controls, even without global cognitive decline (Lee et al. 2010; Salmi et al. 2020; Fallon et al. 2017). These impairments directly affect quality of life: Lawson et al. (Lawson et al. 2016) found that mild cognitive impairment (MCI), often characterized by WM dysfunction, predicted poorer outcomes, and Fan et al. (Litvan et al. 2012) reported that PD-MCI patients had markedly worse quality of life than cognitively intact individuals.

Despite growing recognition of cognitive impairment in PD, accurate diagnosis and monitoring remain difficult (Litvan et al. 2012). Traditional neuropsychological tests are time-consuming, burdensome, and lack the temporal resolution to detect subtle neural changes (Grissmann et al. 2017). Early detection is further hindered by symptom overlap with normal aging, reducing diagnostic sensitivity (Beach et al. 2017). Cognitive decline in PD is also heterogeneous, with wide variation in onset, severity, and progression, limiting the utility of standardized assessments for diagnosis and tracking (Aarsland et al. 2021; Carceles-Cordon et al. 2023). Thus, there is an urgent need for reliable biomarkers to predict and measure cognitive decline, thereby supporting the development of disease-modifying therapies (Aarsland et al. 2021). Physiological measures such as EEG and pupillometry have been used to address limitations in the diagnosis of cognitive impairment in PD (Kahya et al. 2018). EEG provides millisecond-level resolution to capture rapid neural dynamics during WM and can reveal subtle processing deficits (Marsicano et al. 2024), while pupillometry indexes autonomic and cognitive processes such as effort and attention, which are sensitive to WM load and decline (Kahya et al. 2018; Unsworth and Robison 2017; Borys, et al. 2017). Most studies, however, rely on unimodal data, isolated features, or conventional statistics that may miss complex relationships (Grissmann et al. 2017; Unsworth and Robison 2017; Annanmaki et al. 2017; Carmona Arroyave et al. 2019). Machine learning (ML) can capture nonlinear patterns in EEG and pupillometry, and early work shows promise for classifying cognitive states and detecting PD-related abnormalities during WM tasks (Oh et al. 2018; Altham et al. 2024). Still, existing pipelines often lack interpretability, robustness, and clinical generalizability, and few provide standardized multimodal integration, wide-range feature extraction, or models that support post-hoc mapping of retained feature contributors back to specific physiological features and the cognitive task stages in which they occur, enabling mechanistic interpretation of group differences (Altham et al. 2024; Shokrpour et al. 2025).

## Method

### Data collection and behavior data analysis

**Fig. 1:**
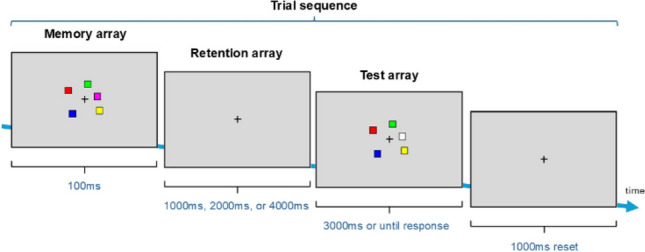
Graphical image of the sequence of tasks from the WM-CDT. Participants first viewed colored squares, followed by different delay periods. This was followed by a test display in which participants responded whether the test display matched the memory display in terms of color (Pashler 1988)

EEG and pupillometry were recorded during a visuospatial WM Change Detection Task (CDT) (Birkholz 2024). On each trial, participants viewed 2–5 colored squares, followed by a variable delay (1000, 2000, or 4000 ms), and then a test display requiring a button press to indicate whether the array matched the original or contained a color change (probability of change = 0.5; see Fig. 1). EEG was collected from 64 scalp electrodes using a BioSemi ActiveTwo system (512 Hz, 0.01–100 Hz bandpass), with additional mastoid and EOG electrodes for artifact monitoring. Data from 68 participants were analyzed (PD: n =

35; HC: n = 33). EEG was segmented into 1195 ms epochs (195 ms pre- to 1000 ms post-memory array onset), baseline-corrected, and preprocessed with independent component analysis (ICA). Pupillometry was embedded in the LAREA channel (an auxiliary ERPLAB channel for time-locking external signals), enabling synchronized feature extraction alongside EEG. ICA was used to identify and remove artifact-related components, including eye blinks, saccades, and muscle activity, based on a combination of component topography, time course, and power spectra, following established EEGLAB guidelines. Components reflecting ocular artifacts were identified by correlating them with EOG channels and by their characteristic frontal scalp distributions and were removed before further analysis. For pupillometry, blink periods were detected and interpolated using standard pupil preprocessing procedures, and trials with excessive signal loss was excluded to ensure a reliable estimation of pupil-based features. WM capacity was estimated with a modified Pashler’s k formula (Pashler 1988), and group differences were tested with repeated-measures ANOVA (Birkholz 2024).

To capture stage-specific processing, EEG and pupillometry features were grouped into task-condition cells defined by memory stage (encoding vs. retrieval), set size (2–5 items), delay (1000, 2000, or 4000 ms), and response accuracy (correct vs. incorrect). This yielded 48 task-relevant task-condition cells, allowing fine-grained alignment of features with specific cognitive demands of the WM-CDT. This task-condition cell structure is central to the pipeline's interpretability: by retaining the correspondence between each feature and its originating task stage, the framework enables post-hoc mapping of retained feature contributors back to specific physiological signals occurring at specific moments during the working memory task, enabling post-hoc investigation of the neural and autonomic dynamics underlying group differences.

### Machine learning pipeline

We used our custom MATLAB toolbox to extract 108 EEG and pupillometry features across ten categories (Terry et al. 2026). The toolbox preserves temporal alignment with task events and focuses on physiologically meaningful markers identified in prior literature (Lee et al. 2010; Grissmann et al. 2017; Kahya et al. 2018; Borys, et al. 2017; Annanmaki et al. 2017; Carmona Arroyave et al. 2019; Terry et al. xxxx; Anjum et al. 2024). For example, frontal alpha asymmetry (FAA) was included as it.

reflects altered frontal alpha power linked to executive control deficits in PD (Lee et al. 2010; Grissmann et al. 2017; Bokura et al. 2005), while pupil diameter variability was included as a marker of cognitive effort during WM tasks (Kahya et al. 2018; Unsworth and Robison 2017). Table 1 lists all extracted features by category.

**Table 1:** All 108 EEG and Pupillometry features across ten categories used in the feature extraction toolbox

Category (*n*)	Features
Time domain (21)	Mean amplitude, standard deviation of amplitude, peak-to-peak amplitude, root mean square amplitude, variance of amplitude, zero crossings, skewness, kurtosis, maximum peak amplitude, minimum peak amplitude, minimum signal value, maximum signal value, signal latency, area under the curve, line length of waveform, signal energy, signal power, noise power, signal-to-noise ratio, autocorrelation at l
Hjorth parameters (4)	Hjorth activity, hjorth mobility, hjorth complexity, hjorth complexity/mobility ratio
Frequency domain (19)	Delta band power, Theta band power, Alpha band power, Beta band power, Gamma band power, Relative band power, Delta peak frequency, Theta peak frequency, Alpha peak frequency, Beta peak frequency, Gamma peak frequency, Alpha/Beta power ratio, Theta/Beta power ratio, Alpha/Theta power ratio, Gamma/Beta power ratio, Mean frequency, Median frequency, Petrosian fractal dimension, Burst percentage
Pupillometry (15)	Mean pupil diameter, Standard deviation of pupil diameter, Minimum pupil diameter, Maximum pupil diameter, Range of pupil diameter, Variance of pupil diameter, Mean pupil velocity, Mean pupil acceleration, Blink rate, Blink durations, Mean blink duration, Task-evoked pupillary response latency, Task-evoked pupillary response peak amplitude, Half recovery time of pupil response, Area under the curve of task-evoked pupillary response
ERP components (12)	N100 amplitude, N100 latency, N200 amplitude, N200 latency, P200 amplitude, P200 latency, P300 amplitude, P300 latency, Average N200 amplitude, Average P300 amplitude, Component amplitude ratio, P300 variability
Phase and synchrony metrics (10)	Theta synchrony, Alpha desynchronization, Phase difference, Phase synchrony, Phase resetting index, Alpha band coherence, Beta band coherence, Phase locking value, Phase locking value mean, Cross-channel coupling
Entropy and complexity (7)	ERP entropy, Tsallis entropy, Hurst exponent, Teager energy operator signal, Mean Teager energy operator, Global field power, Root mean square of successive differences
Spike and waveform Features (5)	Waveform sharpness, Spike frequency, Spike amplitude, Spike/slow wave amplitude ratio, Slow wave activity
Asymmetry and indices (3)	Frontal alpha asymmetry, Prefrontal asymmetry index, Phase- amplitude coupling
Spectral features (12)	Spectral centroid, Spectral entropy, Spectral flatness, Spectral roll-off frequency, Spectral kurtosis, Spectral variability, Spectral edge frequency (95%), Event-related spectral perturbation, Power spectral density (PSD) slope, PSD skewness, PSD kurtosis, Cumulative spectral power

The toolbox extracted features within task-relevant task-condition cells for all participants, preserving the task-condition cell-by-feature structure and temporal dynamics of each trial. Channel-specific measures were computed at electrodes designated by prior literature, while global features were derived from the full signal within each task-condition cell. The same task-condition cell loop was applied across participants, ensuring comparable feature matrices that could then be input into ML models to identify not only which features were most predictive, but also when they occurred. By aligning features with cognitive task stages, the toolbox facilitated the development of interpretable models that can help link EEG and pupillary signals to underlying processes.

Each participant contributed one feature matrix of dimensions 108 features × 48 task-condition cells, yielding 68 feature matrices in total: 35 from PD participants and 33 from HC participants. For classifier input, each participant's 108 × 48 feature matrix was collapsed into a single 108-length feature vector by averaging each feature's value across the 48 task-condition cells (omitting missing values). This produced one observation per participant (68 total; 54 in the training pool, 14 in the hold-out set). PCA and recursive elimination were subsequently applied to this 108-feature, participant-level representation, not to the original 48-cell structure. This aggregation step was necessary given the sample size available for training: flattening the full 108 × 48 structure would yield a feature space more than 96 times larger than the 54-participant training pool, creating a severe small-sample, high-dimensional (P ≫ N) problem in which the model would have substantial opportunity to fit noise rather than generalizable signal, and cross-validated performance estimates would become considerably less trustworthy. Averaging across the 48 task-condition cells reduces the representation to a scale the available sample can more reasonably support, consistent with the sample-size considerations underlying the choice of classifier discussed further in the Discussion. The original, un-aggregated 108 × 48 structure was retained alongside the classifier input to support interpretability: for each of the top-ranked features identified by the classification pipeline, the specific task-condition cell contributing most strongly to that feature's between-group separation was identified from this preserved bin-level data Supplementary (Table 1). This cell-level mapping traces each feature's discriminative signal back to where it concentrated within the task structure, rather than describing a separate process from what the classifier used. A hold-out set was constructed by withholding 20% of participants (n = 14; 7 PD, 7 HC) prior to any model training, feature selection, or preprocessing. This ensured evaluation of participants who were entirely unseen and provided an unbiased estimate of generalization performance. All feature matrices for a withheld participant were excluded, ensuring that no participant contributed data to both the training and evaluation sets and eliminating the risk of data leakage. The remaining 54 participants (28 PD, 26 HC) formed the training pool. See Table 2 for a full breakdown of the dataset's composition (Fig. 1).

**Table 2:** Dataset composition and train/hold out partition summary

Category	PD	HC	Total
All Participants	35	33	68
Training participants (80%)	28	26	54
Hold out participants (20%)	7	7	14
Feature matrices per participant	1(108x48)	1(108x48)	68

**Fig. 2:**
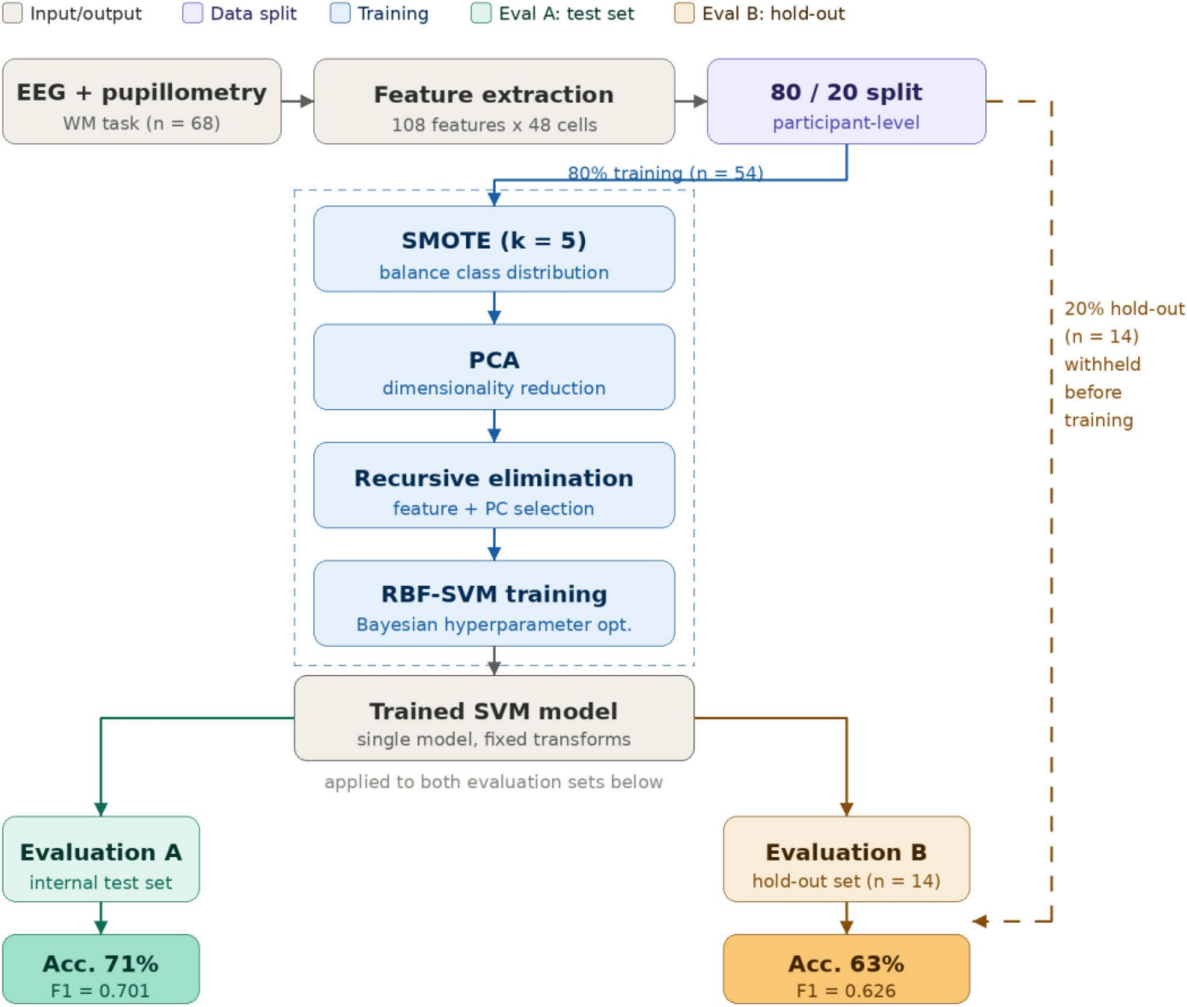
Overview of the ML pipeline developed for classifying PD using EEG and pupillometry data. The process begins with feature extraction using a standardized, temporally aligned toolbox that captures a broad range of neural and physiological features during a WM-CDT. Dimensionality reduction is performed using PCA and automated elbow point detection, followed by RE to identify the most informative components. The optimized feature set is then used to train an SVM-RBF classifier, which is validated using test and holdout datasets

To prevent information leakage between model development and evaluation, all data preparation and model-selection steps were nested within a fivefold cross-validation procedure applied to the 54-participant training pool (28 PD, 26 HC); the 14-participant hold-out set was withheld from every step described below and used only once, for final evaluation. Within each of the five outer folds, the training pool was split into a fold-training portion and a fold-validation portion. Synthetic minority oversampling technique (SMOTE) was applied to the fold-training portion only, generating synthetic minority-class samples to correct the class imbalance within that fold; the corresponding fold-validation portion was left untouched by SMOTE. In the context of a small training set (n = 54 total matrices), even a modest imbalance of this kind has been shown in some small, high-dimensional datasets to bias the decision boundary toward the majority class (Blagus and Lusa 2013), though the size of this effect depends on the specific dataset and classifier. As a precautionary measure against this risk, SMOTE was applied within each cross-validation fold, as described above. Principal component analysis (PCA) was then fit on the SMOTE-balanced fold-training portion, using an automated elbow method to determine the number of retained components, followed by recursive elimination (RE) to further prune components and features, both performed within the fold-training portion only. Bayesian optimization was used to tune the support vector machine with radial basis kernel function (SVM-RBF) hyperparameters (BoxConstraint and KernelScale) within this same fivefold cross-validation loop. The reported test-set accuracy (71%, F1 = 0.701) reflects the average performance across the five fold-validation portions of this nested procedure. Once the optimal pipeline configuration (PCA components, RE-selected features, and SVM hyperparameters) was finalized, SMOTE, PCA, and RE were refit once on the entire 54-participant training pool, and the final model was retrained on this full pool and evaluated once on the untouched hold-out set (63% accuracy, F1 = 0.626), providing an unbiased estimate of generalization performance. Model performance was evaluated using accuracy, F1-scores, and confusion matrices. Final validation was conducted on a hold-out set using the same metrics. Figure 2 illustrates the full pipeline, and the top features identified by RE were mapped back to their temporal task-condition cells via the toolbox’s alignment, clarifying when during the task the most discriminative features occurred.

As a supplementary, exploratory validation step following the primary univariate screening, we conducted spectral-parameterization analysis using FOOOF (Fitting Oscillations & One-Over-F) on the alpha-band spectral-ratio features (alpha/theta, alpha/beta) that emerged as top-ranked in the classification pipeline. This analysis was not part of the main feature-extraction or classification pipeline; rather, it was performed post-hoc to test whether group differences in these spectral-ratio features reflected genuine periodic (oscillatory) alpha-band changes, as opposed to differences in aperiodic (1/f) background activity, which FOOOF explicitly separates. Group comparisons (PD vs. HC) on the resulting FOOOF-derived parameters were conducted using Welch's t-tests (unequal variance), with effect sizes computed as pooled Cohen's d, and p-values corrected for multiple comparisons using the Benjamini–Hochberg false discovery rate (FDR) procedure. Only parameters with at least 10 valid observations per group were retained for statistical testing.

## Result

Behavioral analysis revealed that overall WM capacity was lower in PD participants compared to HCs (F(1,67) = 4.59, p = 0.036, η^2^ = 0.064). Both groups, however, showed a paradoxical improvement in performance with increasing retention intervals (F(2,134) = 7.001, p = 0.001, η^2^ = 0.095), such that capacity estimates were higher at 2000 ms and 4000 ms than at the shortest delay. The group × delay interaction was not significant (F(2,134) = 0.286, p = 0.752, η^2^ = 0.004). Simple effects analysis revealed that the only statistically significant group difference emerged at the 1000 ms delay (p = 0.015), where PD participants performed worse than HCs; however, no differences were found at the longer delays (2000 ms: p = 0.102; 4000 ms: p = 0.087). Behavioral WM capacity results are reported from the parent study's full participant sample (N = 70), using a mixed ANCOVA controlling for depression, anxiety, and fatigue covariates (BDI, STAI, MFI, FSS) (Birkholz 2024). This sample is larger than the n = 68 sample used in the present classification analysis, because a subset of participants did not have sufficient EEG/pupillometry trial data to support reliable feature extraction and were therefore excluded from the ML pipeline despite having usable behavioral data.

After feature extraction, univariate screening of all 108 features revealed small-to-moderate group differences across multiple EEG and pupillometric measures. Effect sizes (Cohen’s d) were generally modest, and few features survived FDR correction, consistent with a diffuse cognitive phenotype rather than a small number of highly discriminative biomarkers. Full univariate statistics are provided in Supplementary Table 3. Effect sizes and p-values for two features could not be computed and are reported as not available in the supplementary table. Specifically, global field power exhibited near-zero between-participant variance after task-aligned segmentation and normalization, resulting in effectively constant values across subjects. Phase difference was undefined because stable phase estimation requires consistent oscillatory power and phase locking across trials, which were not uniformly present across participants and task-condition cells in this dataset. Importantly, such features were effectively downweighted during dimensionality reduction, ensuring that features with minimal variance or unreliable estimates did not meaningfully influence the multivariate classification model. To assess whether the top seven selected features show consistent PD-versus-HC patterns independent of the full-sample screening, we additionally compared PD and HC participants within the 14-participant hold-out set alone (7 PD, 7 HC) at the specific task-condition bin from which each feature was originally selected. None of the seven features reached statistical significance in this subset (Mann–Whitney U, all p > 0.10), which is expected given the substantially reduced power of n = 7 per group relative to the full-sample screening (n = 68). Effect sizes were nonetheless informative: PAI (d =  − 0.66) and alpha/theta ratio (d = 1.05) showed moderate-to-large effects consistent in direction with the full-sample screening, while FAA (d =  − 0.52), ERP latency (d =  − 0.20), alpha/beta ratio (d = 0.01), and half recovery time (d =  − 0.11) showed smaller effects. Delta peak frequency could not be meaningfully evaluated in this subset, as all 14 hold-out participants showed an identical value at the relevant bin. These hold-out comparisons should be interpreted cautiously given the small subgroup size, but they provide a transparent, independent check on the top-selected features, and indicate that full-sample univariate significance does not guarantee detectable group differences in small independent subsets — underscoring the need for larger, externally validated cohorts to confirm these candidate biomarkers.

The elbow point for the PCs occurred at 20 components, indicating that additional components beyond this point contributed minimally to the overall variance structure. Similarly, when evaluating feature importance based on the summed absolute PCA loadings, the elbow point was identified at 22 features. These results suggest that the top 20 principal components and the top 22 original features capture the most critical information for distinguishing between PD and HC groups in WM-CDT. RE found that the model achieved its best performance with 14 principal components and the top 7 PCA-weighted features, yielding an accuracy of 71%, precision of 0.679, recall of 0.669, and an F1 score of 0.701 on the test set.

To validate the model's robustness, we systematically reduced the number of PCs from 13 to 1 while keeping the top 7 features fixed. Performance declined as fewer PCs were used, confirming that 14 components and 7 features provided the most effective and generalizable representation for distinguishing PD from HC in the WM-CDT. The final SVM model, tested on the hold-out set, achieved 63% accuracy with a precision of 0.632, recall of 0.619, and F-score of 0.626. To assess whether the learned feature representation captured group-level cognitive signal beyond chance, we performed a participant-level permutation test on balanced accuracy using the cross-validated test-fold predictions (the same predictions underlying the 71% test-set accuracy reported above). The observed balanced accuracy was 0.701. This value exceeded the permutation distribution (10,000 permutations; CI = permutation p = 0.0013), indicating that the learned multimodal feature representation captured statistically significant group information in the test-fold evaluation. This permutation analysis was not separately repeated on the smaller, hold-out set.

The seven most discriminative features were frontal alpha asymmetry (FAA), prefrontal alpha index (PAI), alpha/theta ratio, ERP latency, alpha/beta ratio, pupillary half recovery time, and delta peak frequency. These features emerged in task-condition cells with the greatest group-level variance, most often during retrieval under high load (set sizes 3–5), on incorrect trials, and with longer delays (2000–4000 ms) Supplementary (Table 3). This pattern raises the possibility of a behavioral-performance confound: if PD and HC participants differed systematically in the number or distribution of incorrect trials, the classifier could be partly learning error frequency or trial-count reliability rather than neurophysiological disease signatures specifically. This possibility is discussed further in the Limitations. Notably, while behavioral performance converged between groups at the longer delays, the most discriminative physiological features were observed in these intervals, suggesting that late-stage, high-effort memory processes provide the clearest markers of group separation.

**Table 3:** Present pipeline in the context of published EEG-based PD classification studies

Study	Modality/features	Classifier	N	Accuracy	Other evaluation metric
Present study	EEG + pupillometry	SVM-RBF (Test set)	54	71%	0.701
Present study	EEG + pupillometry	RBF + SVM (Hold out)	14	63%	0.626
Bera et al. (2025)	Resting EEG,	SVM (Test Set)	88 (subj. depen.)	82–94%	–
Bera et al., (2025)	Resting EEG	SVM (Hold out)	88 (subj. indep)	68%	–
Anjum et al. (2024)	Resting EEG, LPC features	PC + ML ensemble	106	85.7%	AUC = 0.85
Oh et al. (2018)	Resting EEG	13-layer CNN	40	88.3%	Sens. 84.7%
Altham et al. (2024)	EEG, multi-modal review	RF (best reported)	40	91%	AUC = 0.98
Srinivasan et al. (2024)	Voice signals	CNN/FMM + SMOTE	195	98%	F1 = 0.97
Alijalal et al. (2022)	Resting EEG, CSP + Entropy	SVN/KNN	50	75–88%	–

Present study. Test-set = internal validation; hold-out = strictly independent participant-level set, never seen during training

## Discussion

The present pipeline successfully demonstrates that task-aligned, multimodal feature extraction combined with structured dimensionality reduction enables identification of which physiological features contribute most strongly to PD vs. HC classification and precisely when during the working memory task those differences emerge. This is a meaningful advance over prior approaches, and one that directly addresses a gap in the field: cognitive impairment in PD is heterogeneous, temporally dynamic, and distributed across neural and autonomic systems, yet most existing EEG and pupillometry studies rely on isolated features or temporally averaged signals, thereby collapsing the task structure critical for mechanistic interpretation. The novelty of the present framework lies in its standardized, task-aligned, multimodal feature-extraction strategy, which preserves the temporal structure of working memory processing while enabling integration of EEG and pupillometry data. By organizing features within predefined task-condition cells, the pipeline provides a principled means of asking not only whether groups differ, but also when during the task those differences emerge and which physiological systems drive them. This physiological sensitivity is particularly notable given how limited the behavioral separation between groups was in this cohort. Overall WM capacity was significantly lower in PD participants (F(1,67) = 4.59, p = 0.036), but this effect was reliable only at the shortest retention interval (1000 ms) and disappeared at 2000 ms and 4000 ms, with no significant group × delay interaction. In other words, standard behavioral measures of WM performance provided only a narrow and inconsistent window into group differences. Against this backdrop, the classifier's ability to distinguish PD from HC participants with 71% test-set accuracy using EEG and pupillometry features suggests that physiological signals may carry information about group membership that is not reliably captured by overt task performance alone. This is consistent with the broader rationale for the present approach: rather than replacing behavioral assessment, task-aligned physiological features may offer a more sensitive window into cognitive dysfunction in PD, particularly in cases where behavioral deficits are subtle, inconsistent, or not yet clinically apparent.

Within this context, interpretability is defined in terms of feature origin, task alignment, and post-hoc traceability rather than at the level of individual model parameters. The key interpretable property of this pipeline is its ability to map retained feature contributors back to specific physiological features occurring at specific moments during the working memory task: by retaining the correspondence between each feature and its originating task-condition cell, the framework enables post-hoc identification of which signal domains, task stages, and cognitive contexts contributed most to group separation. This allows mechanistically grounded hypotheses about the underlying neural dynamics to be generated directly from the model output — a capability that purely predictive approaches, such as deep networks or ensemble methods, do not provide, as their decision processes cannot be meaningfully related to neurophysiology or cognitive theory. Although PCA mixes features and the SVM-RBF does not yield directly interpretable weights, interpretability is preserved by mapping the retained principal components back to their original contributing features using summed absolute loadings and by maintaining temporal alignment throughout the pipeline. This approach is consistent with established frameworks distinguishing post-hoc traceability from model-parameter transparency as a legitimate and clinically relevant form of interpretability (Lipton 2018; Barnett, et al. 2024). In the next section, we demonstrate this aspect of the pipeline by walking through the top-ranked features and offering provisional interpretations to illustrate the model's potential utility.

A related design choice worth discussing is the use of SMOTE to address class imbalance in the training pool. Given the relatively small imbalance observed (28 PD vs. 26 HC, a 7% difference), it is possible that SMOTE provided limited practical benefit, or that simpler alternatives, such as class-weighting or no rebalancing at all, would have performed comparably. This study did not directly compare classification performance across rebalancing strategies (no rebalancing, class-weighting, SMOTE), so the marginal contribution of SMOTE specifically cannot be determined from the present results. SMOTE was applied here as a precautionary measure, consistent with prior recommendations for small, high-dimensional classification tasks, but future work should conduct this comparison directly to establish whether class-rebalancing is necessary at this level of imbalance, or whether the reported performance is robust to this choice.

Beyond interpretability, the choice of an SVM-RBF was also deliberately suited to the scale of the present data. With 108 extracted features and a sample of 68 participants, this study operates in a small-sample, high-dimensional regime in which more complex or data-hungry approaches, such as deep neural networks or large ensemble methods, are prone to overfitting and typically require substantially larger training sets to generalize reliably. SVMs are comparatively well-suited to this regime: the margin-maximization objective and kernel-based approach to nonlinear class boundaries allow the SVM-RBF to model complex decision surfaces without the large parameter counts that make deep learning approaches difficult to train reliably at this sample size. The selection of SVM-RBF over more recent architectures therefore reflects a deliberate match between model complexity and dataset scale, rather than a default reliance on convention. As larger, multi-site cohorts become available, future work should benchmark this feature set against more current or higher-capacity algorithms, such as gradient-boosted trees or deep learning architectures, to determine whether additional model complexity yields meaningful gains in classification performance once sample size is no longer a limiting factor.

The pipeline's hold-out accuracy of 63% and F1 of 0.626 are best understood by comparison to the most methodologically parallel published work. (Bera et al. 2025) evaluated SVM classification on resting-state EEG reporting accuracy of 82–94% under subject-dependent evaluation and 68.09% under strict subject-independent evaluation — a result directly comparable to the present hold-out, which was obtained under equivalent participant-level separation. The fact that the present pipeline achieves a similar floor to that of the subject-independent resting-state SVM, despite targeting the substantially harder problem of task-evoked cognitive classification in a small, heterogeneous cohort, suggests the pipeline is performing at the level supported by this validation approach and signal type.

Broader comparisons across Table 3 should be interpreted holistically rather than as direct benchmarks, for three reasons. First, studies reporting 88–98% accuracy (Oh et al. 2018; Altham et al. 2024; Aljalal et al. 2022) predominantly use resting-state EEG or voice signals, which capture well-established motor signatures of PD that are neurologically more robust and less variable than the task-evoked cognitive signals targeted here. Second, most published benchmarks rely on subject-dependent evaluation, which is known to overestimate performance on truly unseen participants. Third, sample sizes, preprocessing strategies, and validation designs vary substantially across studies, making numerical comparison uninformative without accounting for these factors (Shokrpour et al. 2025). Taken together, the present results are consistent with the range expected for strict participant-level validation on task-based cognitive EEG in a small clinical cohort, onand the permutation-validated test-fold balanced accuracy of 0.701 confirms that the pipeline captures genuine group-level signal rather than noise in cross-validated evaluation.

### Feature level insights

The RE analysis identified several features that differentiated PD from HC. As shown in Table 2, task-condition cells are defined by task phase, set size, accuracy, and delay length, thereby linking features to specific cognitive contexts. The full behavioral results are reported in detail in the original study on which this ML model was trained and tested (Birkholz 2024). Still, several general findings are relevant for interpreting feature-level patterns. We emphasize that these findings demonstrate the pipeline’s potential rather than definitive biomarkers. Accordingly, feature-level interpretations are presented to illustrate how the pipeline can generate mechanistically grounded hypotheses when linked to task structure and prior behavioral findings, rather than to assert causal or diagnostic conclusions. Furthermore, while PCA components are linear combinations of features, interpretation in this study was not based on individual components themselves. Instead, we examined which original, physiologically defined features consistently contributed to retained components and survived RE, grounding interpretation in the original feature space rather than in abstract PCA axes. This strategy preserves interpretability at the feature level by maintaining direct correspondence to known neurophysiological measures, while still benefiting from dimensionality reduction to mitigate redundancy and noise. This interpretation strategy is supported by univariate statistics, which revealed small-to-moderate effect sizes across multiple features rather than a single dominant discriminator, reinforcing the need for a multivariate framework.

Among the top-ranked features, frontal asymmetry measures emerged at later task stages and under higher cognitive load. PAI emerged during retrieval at 2000 ms, while FAA emerged at 4000 ms, suggesting a shift from fine-grained prefrontal control to broader frontal recruitment under higher demand (Ghaddar and Naoum-Sawaya 2018). Behavioral results supported this interpretation: WM capacity was significantly lower in PD at 1000 ms but not at longer delays. Performance improved from 1000 to 2000 ms in both groups, with a plateau in HCs and a slight dip in PD. This suggests that the shortest delay may not allow sufficient time for response preparation, whereas additional time enables compensatory processes. However, not all studies report this pattern. Fallon et al. (Fallon et al. 2017) found that PD patients showed persistent deficits at longer delays, with recall precision degrading more quickly over time, regardless of current dopaminergic medication status (ON/OFF). This discrepancy likely reflects task differences; our CDT involves discrete change detection with variable set sizes, whereas Fallon’s continuous reproduction task was more sensitive to gradual memory decay. Together, these findings suggest PD-related WM impairments may arise from either early encoding/retrieval inefficiency or accelerated decay, depending on task demands and response format (Ye et al. 2022). Importantly, the emergence of FAA and PAI at later task stages underscores the value of temporally aligned feature extraction, as these effects would be obscured in analyses that collapse across task phases or delay conditions.

Oscillatory ratios also differentiated groups. Alpha/theta and alpha/beta ratios showed moderate effect sizes (α/θ: Cohen’s d = 0.779, p = 0.00279; α/β: Cohen’s d = 0.405, p = 0.1060). Alpha/theta, emerging during encoding, and alpha/beta, observed during retrieval, were both elevated in PD, consistent with altered attentional control and inhibitory processes (Ye et al. 2022). The improvement from 1000 to 2000 ms and subsequent stabilization parallels these oscillatory dynamics, suggesting they may support compensatory performance (Zhu et al. 2019; Klimesch 2012).

To further validate the spectral-ratio features, we performed an additional spectral-parameterization analysis using FOOOF to separate periodic oscillatory components from the aperiodic 1/f background. This analysis showed group differences in alpha peak parameters, including peak height and center frequency, indicating that differences in the aperiodic slope did not solely drive the observed α/θ and α/β elevations. Additionally, FOOOF-derived features revealed large effect sizes for alpha peak height at both frontal and posterior sites (PostFz_AlphaHeight: Cohen’s d = 0.84, p = 0.0021; PostCz_AlphaHeight: Cohen’s d = 0.84, p = 0.0018; PostFz_AlphaHHeight: Cohen’s d = 1.01, p = 0.0088), demonstrating that group differences reflect genuine alterations in alpha oscillatory magnitude rather than aperiodic slope alone. Additionally, aperiodic offset differences (PrePz_offset: Cohen’s d = 0.65, p = 0.0093) indicate altered broadband excitation–inhibition balance in PD, further motivating the inclusion of spectral parameterization analyses to separate oscillatory from aperiodic contributions. Although individual alpha frequency was not explicitly normalized during feature extraction, shifts in alpha peak frequency are themselves well documented in PD. They may reflect disease-related alterations rather than methodological bias (Ye et al. 2022; Zhu et al. 2019; Klimesch 2012). These findings suggest that spectral-ratio features capture meaningful oscillatory changes and highlight opportunities for future refinement through explicit IAF normalization.

Time-sensitive features added further evidence where signal latency, defined in our toolbox as the time of maximum positive deflection within a task-condition cell, was prolonged in PD during retrieval at 2000 ms (Annanmaki et al. 2017; Demirayak et al. 2023) with a small-to-moderate effect (Cohen’s d = 0.213, p = 0.4122). Although component-agnostic, this measure can be mapped to ERP epochs and electrode sites for comparison with canonical markers (e.g., P200, P300). To complement this, ERP-specific latencies (N100, P200, P300) were also extracted, directly targeting well-characterized cognitive stages.

In addition, pupillary half recovery time was reduced in PD at 4000 ms, indicating altered autonomic recovery dynamics during sustained task engagement. This feature exhibited a small effect size (d = 0.117, p = 0.6552), reflecting substantial inter-individual variability and reinforcing the idea that autonomic features act as complementary rather than primary discriminators. Prior work has shown that pupillary recovery timing reflects locus coeruleus–norepinephrine signaling and task-dependent regulation of arousal and autonomic control, rather than a single cognitive construct (Kahya et al. 2018; Unsworth and Robison 2017). Within this framework, reduced recovery time in PD may reflect compensatory autonomic adjustments engaged under prolonged task demands (Kahya et al. 2018; Unsworth and Robison 2017).

Taken together, these findings suggest a tentative compensatory sequence in which PD participants show deficits under high temporal demands, followed by oscillatory, frontal, and autonomic adjustments that help sustain performance at longer delays. The pattern of Cohen’s d values across features demonstrates that cognitive dysfunction in PD is characterized by multiple moderate-to-large, yet distributed, effects, rather than a single dominant marker, providing empirical justification for the multivariate, task-aligned approach adopted here. Importantly, this interpretation is intended to demonstrate how temporally aligned, multimodal feature extraction can contextualize ML-derived feature rankings within known cognitive and neurophysiological frameworks. Rather than relying on a single dominant marker, the results indicate that discriminative information in PD is distributed across task stages and signal domains within this feature set; whether this distributed pattern requires multimodal integration specifically, as opposed to a sufficiently rich unimodal feature set, remains to be tested directly. While preliminary, these patterns illustrate the pipeline's utility for hypothesis generation and motivate further validation in larger, clinically stratified cohorts.

As with all aggregate cohort studies, clinical heterogeneity within the PD cohort, including disease duration, motor severity, cognitive status, and dopaminergic medication status, was not explicitly modeled in the present analyses. These factors are known to influence EEG and pupillometric measures and may contribute to within-group variability in the extracted features. Accordingly, the current results reflect aggregate group-level patterns. They should be interpreted as demonstrating the feasibility of a standardized analytic framework rather than isolating effects attributable to specific clinical subtypes, which will be an important focus of future studies with richer clinical phenotyping. Future work incorporating detailed clinical stratification will be critical for determining whether the observed compensatory patterns differ systematically across disease stages, cognitive profiles, or medication states.

### Implications

This study demonstrates that a task-aligned, multimodal EEG and pupillometric feature set can capture distributed cognitive signatures of PD across task stages and signal domains, though whether this specifically requires multimodal integration, rather than a sufficiently comprehensive unimodal feature set, has not yet been directly tested. The pipeline's core strength lies in its ability to provide task-stage traceability of retained feature contributors to specific physiological processes at specific moments in the working memory task— a property that enables mechanistically grounded hypothesis generation rather than treating the model as a black box. The univariate analyses confirm that no single feature dominates group separation; instead, discriminative information is distributed across multiple moderate-effect features spanning oscillatory, frontal asymmetry, and autonomic domains. The multivariate model integrates these distributed signals into statistically above-chance classification performance, with a permutation-validated test-fold balanced accuracy of 0.701 (p = 0.0013), confirming that the learned representation captures genuine group-level cognitive signal in cross-validated evaluation. This is a particularly meaningful result given the small cohort size and the inherent difficulty of task-evoked cognitive classification, and it underscores the value of the task-aligned integration strategy, though the specific added value of combining modalities rather than using either alone has not yet been directly tested.

The pipeline's performance is best understood in the context of what it is designed to do. Rather than maximizing classification accuracy on a well-characterized resting-state signal, it is designed to reveal the temporal and physiological structure of cognitive dysfunction in PD during an active working memory task — a substantially more challenging and less explored problem. The results establish that this is feasible: multimodal task-aligned features, organized within predefined cognitive task stages and reduced to their most informative dimensions, provide statistically reliable group separation even in a small, clinically heterogeneous cohort under conservative participant-level hold-out evaluation. The feature-level insights generated by the pipeline — including the emergence of frontal asymmetry, changes in oscillatory ratios, and differences in autonomic recovery at specific task stages illustrate the framework's capacity to yield targeted, physiologically grounded hypotheses — a capability that positions this pipeline as a practical tool for hypothesis-driven investigation of cognitive dysfunction in PD.

Accordingly, the framework's primary contribution is methodological and mechanistic: it provides a reproducible, standardized platform for examining how multimodal physiological signals relate to working memory dysfunction in PD, with temporal resolution that conventional analyses cannot achieve. With further validation in larger, more diverse cohorts, this approach has the potential to support both biomarker discovery and the development of task-based cognitive monitoring tools to track disease progression and treatment response in PD.

Taken together, the present framework makes three contributions that distinguish it from existing EEG-based ML approaches for PD. First, it introduces a standardized, task-aligned feature-extraction strategy organized within cognitive task-condition cells, enabling systematic comparison across encoding, maintenance, and retrieval stages that prior unimodal or temporally averaged pipelines cannot achieve. Second, it provides post hoc feature traceability, the ability to identify which physiological features contributed most to classification and precisely when during the working memory task those contributions occurred — thereby linking ML outputs directly to neurophysiologically interpretable processes rather than treating the classifier as a black box. Third, it demonstrates that distributed, temporally structured cognitive signatures of PD are detectable under conservative participant-level hold-out validation, establishing that statistically reliable group separation is achievable even in small, heterogeneous cohorts using this task-aligned feature set, though the specific contribution of multimodal (versus unimodal) integration remains untested. These properties position the framework as a principled research platform for cognitive biomarker discovery in PD, with a clear path toward validation in larger cohorts and translation into longitudinal monitoring applications.

## Limitations and future work

While the present pipeline addresses several gaps in existing EEG-based approaches, its generalizability is constrained by the relatively small, single-task cohort and the high dimensionality of the feature space. Although model selection and hyperparameter tuning were confined to the training set and final evaluation was performed on a strictly independent hold-out set, the modest performance on unseen participants indicates that overfitting remains a concern and highlights the need for larger and more diverse datasets. Importantly, the WM-CDT was chosen to probe specific WM processes rather than to maximize diagnostic sensitivity; thus, the extracted features should be interpreted as candidate markers of cognitive dysfunction rather than definitive diagnostic biomarkers.

Interpretation of spectral features is also limited by the frequent absence of clear oscillatory peaks outside the alpha band, which complicates the use of ratio-based metrics and peak counting, particularly for delta and theta activity. To address this, we incorporated spectral parameterization analyses to separate periodic oscillatory activity from the aperiodic 1/f component, improving interpretability and reducing the likelihood that group differences reflect broadband spectral slope rather than true oscillatory changes. Nonetheless, future work should more fully integrate parameterized spectral features and individual alpha frequency normalization to validate oscillatory findings further.

This study does not directly compare EEG-only, pupillometry-only, and combined EEG-pupillometry models, and therefore cannot demonstrate that the multimodal representation provides classification information beyond what either modality alone would provide. Consistent with this gap, the top seven selected features were predominantly EEG-derived (six of seven), with only one pupillometry-derived feature (pupillary half recovery time) retained in the final model. Future work should conduct modality-ablation analyses (EEG-only, pupillometry-only, and combined models under the same pipeline) to directly quantify the incremental value of multimodal integration, and to determine whether pupillometry's limited representation among top features reflects a genuine physiological pattern or a limitation of the current feature set or selection procedure.

Relatedly, this study did not report trial counts per task-condition cell broken down by group, nor document how missing or empty cells were handled during feature extraction, both of which would help rule out the possibility that group differences in trial count or data completeness contributed to the observed feature patterns. We also did not test whether the top-selected features, several of which emerged from incorrect-trial cells, remain discriminative after statistically controlling for behavioral error rate or accuracy. As a result, we cannot currently rule out that some of the reported group differences partly reflect behavioral-performance confounds rather than neurophysiological signal alone. Future work should report per-cell trial counts by group, document missing-data handling explicitly, and test feature discriminability after controlling for behavioral performance to isolate genuine neurophysiological contributions from trial-count or error-frequency artifacts. Additional limitations include clinical heterogeneity within the PD group, including variability in disease duration, cognitive status, and medication effects, which were not modeled at the individual level and may contribute to variability in features. Future studies should explicitly incorporate clinical covariates and examine relationships between model outputs and behavioral measures to link neural features to cognitive performance better. More broadly, future research should prioritize replication across larger, more heterogeneous cohorts, evaluate generalizability across cognitive tasks and recording paradigms, and assess longitudinal sensitivity to disease progression. Collectively, these steps are necessary to determine which features are stable, task-general, and clinically meaningful, and to support the eventual translation of this framework from a mechanistic research tool to a clinically relevant platform for monitoring cognitive dysfunction in PD.

## Conclusion

This study presents a multimodal ML pipeline that integrates task-aligned EEG and pupillometry features using a standardized MATLAB-based extraction toolbox to distinguish individuals with PD from HC during a WM-CDT. By explicitly aligning neural and autonomic features to defined task stages and applying structured dimensionality reduction, the pipeline enables identification of temporally specific and physiologically grounded patterns that reflect distributed, compensatory cognitive processes in PD.

Although classification performance was modest and constrained by sample size, task specificity, and cohort heterogeneity, the results demonstrate that cognitive dysfunction in PD is characterized by temporally structured effects across task phases and signal domains within this feature set; whether this pattern specifically requires multimodal integration, as opposed to a sufficiently rich unimodal feature set, has not been directly tested here. Accordingly, the primary contribution of this work is not a deployable diagnostic classifier, but a principled framework for systematically examining how multimodal physiological signals relate to cognitive function in PD.

Together, these findings establish a foundation for future studies aimed at validating feature stability, expanding to larger and more diverse cohorts, and extending the approach across cognitive paradigms and longitudinal designs. With further development and validation, this framework may ultimately support research efforts to track cognitive changes in PD and to inform the design of clinically meaningful biomarkers.

## Supplementary Information

Below is the link to the electronic supplementary material.

Supplementary file1 (DOCX 20 kb)

## Data Availability

De-identified feature-level data, behavioral metrics, and analysis scripts are available from the corresponding author upon reasonable request. Raw EEG and pupillometry recordings are available from co-authors Dr. Jeffrey S. Johnson or Dr. Samuel A. Birkholz upon reasonable request, subject to institutional and ethical regulations governing human subject research.
