# Supplementary material for: A task-aligned multimodal machine learning framework for studying working memory dysfunction in Parkinson’s disease
Source: Cogn Neurodyn. 2026 Aug 13;20(1):158. doi: 10.1007/s11571-026-10526-z (PMC13469056; doi:10.1007/s11571-026-10526-z)
Supplement: Supplementary file 1 [file 11571_2026_10526_MOESM1_ESM.docx]

Supplementary Material

Table 1

Table 1 Participant-level univariate feature differences between Parkinson’s disease and healthy controls

| Feature | Cohen’s d | p value |  | Feature | Cohen’s d | p value |
| --- | --- | --- | --- | --- | --- | --- |
| Mean amplitude | 0.429 | 0.09934 |  | **Task-evoked related potential (Tepr) latency** | -0.205 | 0.4268 |
| Standard Deviation amplitude | 0.473 |  |  | **Tepr peak amplitude** | 0.424 | 0.09461 |
| Peak to peak | 0.498 | 0.04853 |  | **Half recovery time** | 0.117 | 0.6552 |
| Root mean Square (RMS) amplitude | 0.486 | 0.05961 |  | **Auc tepr** | 0.429 | 0.09959 |
| Zero crossing | 0.000 | 0.9996 |  | **Alpha coherence** | 0.188 | 0.4529 |
| Variance amplitude | 0.370 | 0.1395 |  | **Phase locking value (Plv)** | 0.275 | 0.2928 |
| Skewness | -0.119 | 0.6488 |  | **Prefrontal alpha index (Pai)** | -0.455 | 0.1098 |
| Kurtosis | 0.173 | 0.4986 |  | **Frontal alpha asymmetry (Faa)** | 0.058 | 0.8166 |
| Max peak | 0.424 | 0.09461 |  | **Phase amplitude coupling (Pac)** | 0.429 | 0.09934 |
| Min peak | 0.543 | 0.03107 |  | **Sharpness** | 0.583 | 0.02317 |
| Hjorth activity | 0.370 | 0.1395 |  | **Spike frequency** | 0.166 | 0.5212 |
| Hjorth mobility | 0.202 | 0.4360 |  | **Spike amplitude** | 0.498 | 0.04853 |
| Hjorth complexity | -0.435 | 0.1049 |  | **Spike slow wave ratio** | -0.034 | 0.8934 |
| Hjorth ratio | -0.531 | 0.05504 |  | **Slow wave activity (Swa)** | 0.131 | 0.6200 |
| Delta power | 0.020 | 0.9412 |  | **Cross coupling** | 0.445 | 0.07061 |
| Theta power | 0.577 | 0.02217 |  | **Globa field power (Gfp)** | NaN | NaN |
| Alpha power | 0.734 | 0.00401 |  | **Tsallis entropy** | 0.304 | 0.2458 |
| Beta power | 0.391 | 0.1103 |  | **Beta coherence** | 0.160 | 0.5229 |
| Gamma power | 0.271 | 0.2632 |  | **Phase locking value mean** | 0.275 | 0.2928 |
| Relative band power | 0.230 | 0.3605 |  | **Erp entropy** | 0.219 | 0.3618 |
| Slope PSD | 0.407 | 0.1195 |  | **Hurst exponent** | 0.003 | 0.9922 |
| Delta peak frequency | 0.059 | 0.8188 |  | **Phase difference** | NaN | NaN |
| Theta peak frequency | 1.032 | 0.00014 |  | **Phase synchrony** | 0.275 | 0.2928 |
| Alpha peak frequency | -0.029 | 0.9119 |  | **Phase resetting** | 0.433 | 0.09665 |
| Beta peak frequency | -0.093 | 0.7162 |  | **Teager energy operator (Teo) signal** | 0.296 | 0.2219 |
| Gamma peak frequency | -0.032 | 0.9021 |  | **Mean Teo** | 0.296 | 0.2219 |
| Alpha beta ratio | 0.405 | 0.1060 |  | **Cumulative power** | 0.224 | 0.3925 |
| Theta beta ratio | -0.299 | 0.2516 |  | **Spectral edge frequency (95%),** | 0.157 | 0.5411 |
| Alpha theta ratio | 0.779 | 0.00279 |  | **Mean frequency** | 0.157 | 0.5450 |
| Gamma beta ratio | -0.030 | 0.9065 |  | **Median frequency** | 0.163 | 0.5316 |
| Theta sync | 0.275 | 0.2928 |  | **Interquartile range (Iqr) of amplitude** | 0.466 | 0.06923 |
| Alpha desynchrony | 0.275 | 0.2928 |  | **Line length** | 0.423 | 0.08913 |
| Spectral centroid | 0.157 | 0.5450 |  | **Energy** | 0.367 | 0.1504 |
| Spectral entropy | 0.152 | 0.5649 |  | **Power Spectral Density (Psd) skewness** | -0.050 | 0.8453 |
| Spectral flatness | 0.017 | 0.9468 |  | **Psd kurtosis** | -0.098 | 0.7000 |
| Spectral roll off | 0.147 | 0.5694 |  | **Petrosian fractal dimension** | 0.273 | 0.2967 |
| Spectral kurtosis | -0.098 | 0.7000 |  | **Burst percentage** | -0.040 | 0.8743 |
| Spectral variability | 0.213 | 0.4178 |  | **Root mean square of successive difference (Rmssd)** | 0.423 | 0.08888 |
| Event-related spectral perturbation (Ersp) | -0.371 | 0.1671 |  | **Signal power** | 0.367 | 0.1504 |
| X min | 0.543 | 0.03107 |  | **Noise power** | 0.293 | 0.2261 |
| X max | 0.424 | 0.09461 |  | **Signal-to-noise ratio (Snr) value** | -0.046 | 0.8597 |
| ERP Latency | 0.213 | 0.4122 |  | **Autocorrelation lag1** | 0.230 | 0.3787 |
| Area under curve (AUC) | 0.429 | 0.09959 |  | **N100 amplitude** | 0.394 | 0.1187 |
| Mean pupil diameter | 0.429 | 0.09934 |  | **N100 latency** | 0.238 | 0.3664 |
| Standard deviation of pupil diameter | 0.473 | 0.06392 |  | **N200 amplitude** | 0.128 | 0.6116 |
| Min pupil diameter | 0.543 | 0.03107 |  | **N200 latency** | -0.267 | 0.3045 |
| Max pupil diameter | 0.424 | 0.09461 |  | **P200 amplitude** | 0.547 | 0.03117 |
| Range of pupil diameter | 0.498 | 0.04853 |  | **P200 latency** | 0.234 | 0.3814 |
| variance of pupil diameter | 0.370 | 0.1395 |  | **P300 amplitude** | 0.367 | 0.1479 |
| Pupil velocity mean | 0.583 | 0.02317 |  | **P300 latency** | -0.325 | 0.2207 |
| Pupil acceleration mean | 0.402 | 0.1059 |  | **N200 amplitude average** | 0.441 | 0.09861 |
| Blink rate | 0.231 | 0.3785 |  | **P300 amplitude average** | 0.212 | 0.4152 |
| Blink durations | 0.007 | 0.9792 |  | **Component ratio** | 0.200 | 0.4110 |
| Mean blink duration | 0.007 | 0.9792 |  | **P300 variability** | 0.332 | 0.1803 |
